# Identification of Two Metallothioneins as Novel Inhalative Coffee Allergens Cof a 2 and Cof a 3

**DOI:** 10.1371/journal.pone.0126455

**Published:** 2015-05-11

**Authors:** Ulrike Peters, Karsten Frenzel, Reinhold Brettschneider, Marcus Oldenburg, Cordula Bittner

**Affiliations:** 1 Biocenter Klein Flottbek and Botanical Garden, University of Hamburg, Hamburg, Germany; 2 Institute for Occupational Medicine and Maritime Medicine (ZfAM), University Medical Center Hamburg-Eppendorf, Hamburg, Germany; The Ohio State University, UNITED STATES

## Abstract

**Background:**

Dust of green coffee beans is known to be a relevant cause for occupational allergic disorders in coffee industry workers. Recently, we described the first coffee allergen (Cof a 1) establishing an allergenic potential of green coffee dust.

**Objective:**

Our aim was to identify allergenic components of green coffee in order to enhance inhalative coffee allergy diagnosis.

**Methods:**

A *Coffea arabica* pJuFo cDNA phage display library was created and screened for IgE binding with sera from allergic coffee workers. Two further coffee allergens were identified by sequence analysis, expressed in *E*. *coli*, and evaluated by Western blots. The prevalence of sensitization to recombinant Cof a 1, Cof a 2, and Cof a 3 and to commercially available extract was investigated by ELISA (enzyme-linked immunosorbent assay) respectively CAP (capacity test) screening in 18 sera of symptomatic coffee workers.

**Results:**

In addition to the previously described chitinase Cof a 1, two Coffea arabica cysteine-rich metallothioneins of 9 and 7 kDa were identified and included in the IUIS Allergen Nomenclature as Cof a 2 and Cof a 3. Serum IgE antibodies to at least one of the recombinant allergens were found in 8 out of 18 symptomatic coffee workers (44%). Only 2 of the analysed sera (11%) had reacted previously to the commercial allergy test.

**Conclusions:**

In addition to the previously described Cof a 1 we have identified two further coffee proteins to be type I coffee allergens (Cof a 2 and Cof a 3) which may have a relevant potential for the specific diagnosis and/or therapy of coffee allergy.

## Introduction

Coffee is the most favorite beverage of hundreds of millions people worldwide. Up to twice a year, in coffee growing countries tons of coffee cherries are harvested. After removing their pulps, the green coffee beans are dried by spreading them under the sunlight. Usually, for further processing steps like roasting and/or decaffeinating, the dried beans are shipped worldwide in large coffee containers. About 25 millions of coffee workers are exposed during the coffee processing to irritating and sensitizing dust with several allergens worldwide (i.e. from burlap, cockroach and coffee bean) [1]. Dust from raw coffee beans has been reported to elicit skin, ocular, and respiratory allergic reactions in up to 50% of coffee processing industry workers [2–4].

To date, reliable diagnostics for coffee sensitization are not commercially available. The only commercial allergy test is a serological test based on a mixture of green coffee bean extracts. These native extracts are of limited sensitivity since they differ in antigen concentration and composition due to lack of standardized allergen sources and preparation procedures. This might lead to false-negative test results. Thus the unpleasant and invasive inhalative provocation test, which is not free of risk, is frequently needed to establish the definitive diagnosis. Therefore, there is a need for improving diagnostic tools for allergy to green coffee dust. Diagnostics based on recombinant antibodies can raise test specificity and sensitivity [5,6]. In our previous research we have identified the class III chitinase Cof a 1 on molecular level [7]. There is a need to expand the coffee allergen panel by identifying further relevant allergens which can be added to available diagnostics, i.e. by spiking commercial extracts with recombinant proteins. The aim of the present work was to identify and characterize further allergens of the *Coffea arabica* bean in order to improve coffee allergy diagnostics.

## Methods

### Investigated sera

Sera for IgE immunodetection were obtained from 18 coffee industry workers from a haulage company, a coffee silo, and a decaffeinating company, all complaining about work-related urticaria, rhinitis, conjunctivitis and/or chest tightness during exposure to coffee dust. None of the employees reported symptoms after drinking coffee. All participants signed written informed consent after explanation of the study by the examining physician. Sera were collected and analyzed according to a study protocol after approval of the study by the Ethics Committee of the local Medical Association (Ethik-Kommission der Ärztekammer Hamburg, approval number: PV3968). All volunteers were male and between 29 and 54 years old (average 45 years). The control sera came from 8 subjects (4 females and 4 males with an average age of 37 years) not occupationally exposed to coffee dust (Tab. 1). None of the controls had ever been diagnosed an allergic conjunctivitis, rhinitis and/or obstructive airways disease nor had a known sensitization to common environmental or occupational allergens. All sera had been stored at −20°C until analysis was performed. Sera from two further workers otherwise not included in the study were used for the biopanning procedure.

### Generation of a cDNA library

A cDNA λTriplEx2 library from immature green coffee cherries (*Coffea arabica*) was built as described previously [6]. Briefly, 1 μg of total RNA was used as starting material to generate double-stranded cDNA according to the PCR-based method. After *in vivo* conversion of the λTriplEx2 library into a pTriplEx2 plasmid population and extraction of the recombinant plasmids, 100 μg of plasmid DNA were digested with *Eco*RI/*Xho*I and resulting inserts were ligated into the *Eco*RI and *Xho*I sites of pJufo II (kindly supplied by Professor R. Crameri, Department Molecular Allergology, Swiss Institute of Allergy and Asthma Research, Davos, Switzerland).

### Biopanning procedure

For selection of IgE-binding proteins presented on the phage surface, the phage display cDNA library was screened by a biopanning approach as described elsewhere [7,8]. For screening the phage display cDNA library we used the sera from 2 subjects with work-related symptoms of rhinoconjunctivitis and sensitization to green coffee beans by ImmunoCAP (k70, Thermo Fisher Scientific Inc), which we had identified in a previous study [7]. The sera of these two subjects did not undergo any further analysis in the present study.

### Cloning of two metallothioneins

The cDNA inserts of the selected clones coding for Cof a 2 and Cof a 3 was sequenced using the ABI Prism Big Dye reaction kit (Applied Biosystems, Darmstadt, Germany) and ABI Prism sequencer. Four oligonucleotides containing *Spe* I/enterokinase and *Xho* I cutting sites were designed to flank the open reading frames (ORF).

Cof a 2: Fw: ATATATACTAGTGATGACGACGACAAGCAAGATGTACCCCGAGTTGA; Rev: ATATATCTCGAGtttgcagttgcagggattgc.

Cof a 3: Fw: ATATATACTAGTGATGACGACGACAAGTCGGACAAGTGCGGAAACTG; Rev: ATATATCTCGAGATTGTCACAGGTGCAGTTGACACAGGCGCA (Cutting sites are underlined). The cDNA clone was used in PCR amplification as template. The PCR products were digested with *Spe* I and *Xho* I and inserted into *E*. *coli* protein expression vector pET-41 b(+) (Novagen, Darmstadt, Germany). After confirming the correct sequences of the clone, they were transformed into *E*. *coli* BL21 (DE3) cells (Novagen).

### Sequence analysis

Sequence data were analyzed using the software package DNASTAR 4.05. Protein domains were identified using the ScanProsite tool ExPASy Proteomics Server [9]. The presence of the targeting signal was determined using Target P and Signal P 3.0 [10]. Alignments were calculated with Clustal W2 [11] and visualized by GeneDoc 2.6.002 [12].

### Expression and affinity purification of the recombinant metallothioneins

*E*. *coli* cultures were grown at 37°C in LB (lysogeny broth-medium) with kanamycin to an OD_600 nm_ (optical density _600 nm_) of 0.5–0.8. For native GST (Glutathione S-transferase) affinity purification the culture was transferred onto ice-cold water for 10 min. Expression of the recombinant protein was induced by adding of IPTG (isopropyl-*β*-D-thiogalactopyranoside) to 1 mM and incubation for 3 h at 22°C. The *E*. *coli* cells were harvested and resuspended in ice-cold lysis buffer (4.3 mM Na_2_HPO_4_, 1.47 mM KH_2_PO_4_, 137 mM NaCl, 2.7 mM KCl, pH 7.3) supplemented with 1 mM protease inhibitor PMSF (phenylmethanesulfonylfluoride or phenylmethylsulfonyl fluoride) (Applichem, Darmstadt, Germany). The following steps were performed at 4°C. Cell lysis was carried out adding lysozyme to a final concentration of 0.07 mg/ml and incubation for 30 min on ice, followed by three freeze-thaw cycles (with fluid nitrogen in a 42°C warm water bath) and sonification. The lysate was cleared by three centrifugation steps at 14,000 rpm for 20 min. The post-centrifugation supernatant was incubated with GST-banded resin (Novagen, Darmstadt, Germany) at 4°C for 90 min under rotation. After three washing steps with lysis buffer, recombinant protein was eluted with elution buffer (50 mM Tris-HCl, pH 8.0, 10 mM reduced glutathione). Protein concentration was determined by Bradford assay using bovine serum albumin as standard. Purity of recovered recombinant proteins was verified by SDS-PAGE.

### Immunoblot Analysis

Recombinant proteins were separated by SDS-PAGE and transferred onto ECL membranes (GE Healthcare, Freiburg, Germany). Protein gel blots were saturated with Penta-His antibody (Qiagen, Hilden, Germany) in a dilution of 1/2000. Goat anti-mouse antibody conjugated to horseradish peroxidase (HRP) (Pierce, Bonn, Germany) was used as secondary antibody in a dilution of 1/2000. Super Signal West Dura Extended Duration Substrate (Pierce, Bonn, Germany) was utilized for antigen detection. Blots were exposed to X-ray films.

### ELISA

We determinated the IgE-binding capacity of the previously described purified recombinant protein rCof a 1 as well as of the novel coffee allergens rCof a 2 and rCof a 3 by an allergen-specific ELISA as described elsewhere [7]. The mean OD value (A_405_) plus 3 standard deviations of control sera from 8 unexposed and healthy subjects for the respective antigen was chosen as cutoff point. All sera were tested twice. A result was considered positive (i.e. indicating sensitization) when both tests were positive.

### ImmunoCAP system

To determine levels of total and allergen-specific IgE antibodies in 18 sera from subjects with work-related rhinitis and/or conjunctivitis after coffee dust exposure, an ImmunoCAP assay was performed according to the manufacturer’s instructions of the ImmunoCAP system (Thermo Fisher Scientific Inc, Freiburg, Germany). For detection of allergen-specific IgE antibodies, CAP analysis was carried out with commercially available sponges coupled with a protein extract from green coffee beans (k 70, Thermo Fisher Scientific Inc, Freiburg, Germany). The results were expressed as CAP scores of class 0 to 6. The cutoff value for positivity was 0.35 kUA/L (lower limit of CAP class 1). Total IgE (reported as concentration in kU/L) was detected by ImmunoCAP (Thermo Fisher Scientific Inc, Freiburg, Germany).

## Results

### Identification of the cDNA clones of Cof a 2 and Cof a 3

**Fig 1:**
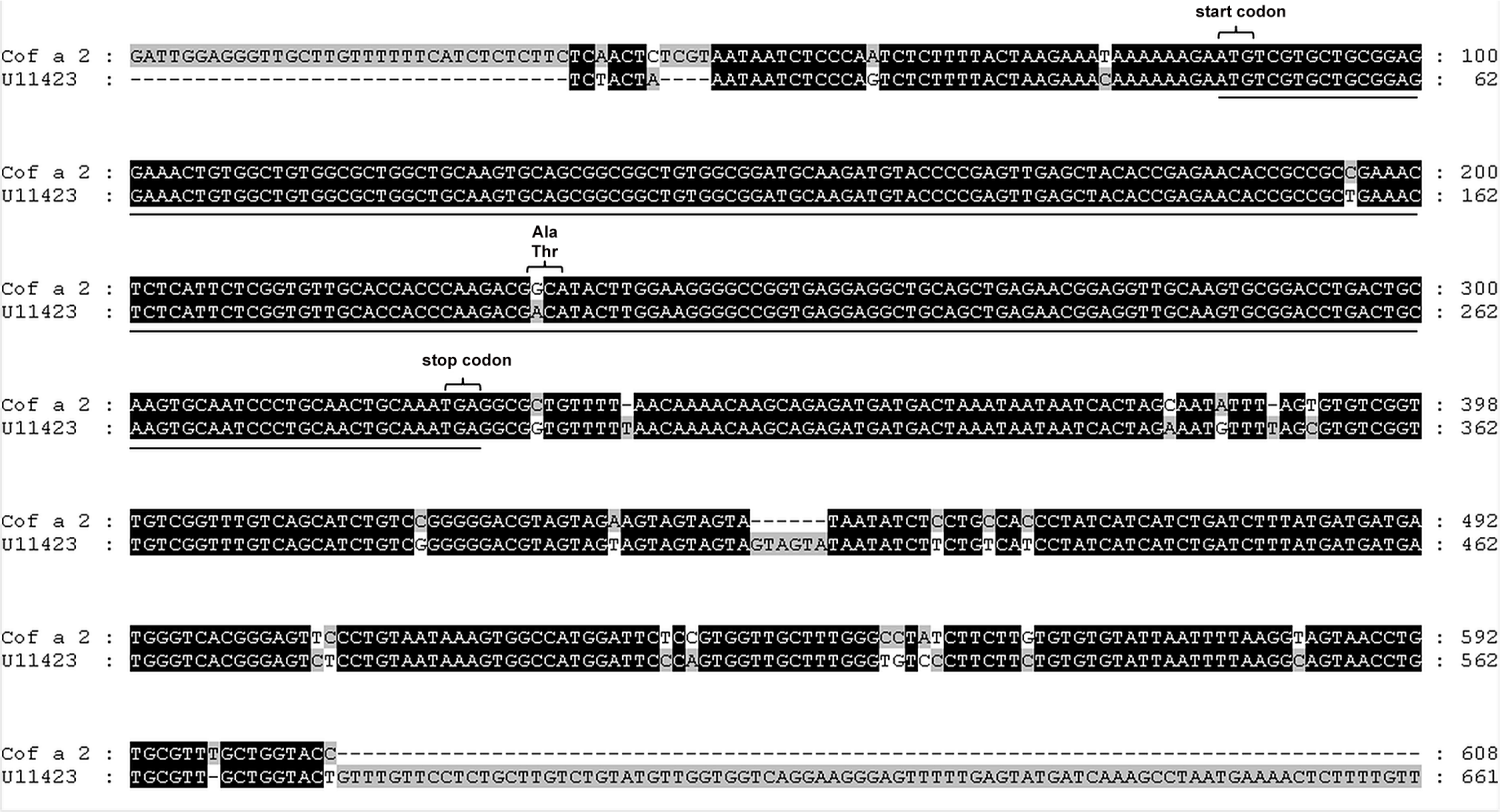
Sequence alignement of Cof a2 cDNA clone and ist homlogue NCBI EST U11423. Homologous regions to the known coffee metallothionein described by Moisyadi et al. ^13^ are highlighted in black. In position 196 and 233 of the protein forming open reading frame a polymorphism in the cDNA sequence is seen (underlined), in position 233–235 there is one polymorphism in the amino acid sequence.

**Fig 2:**
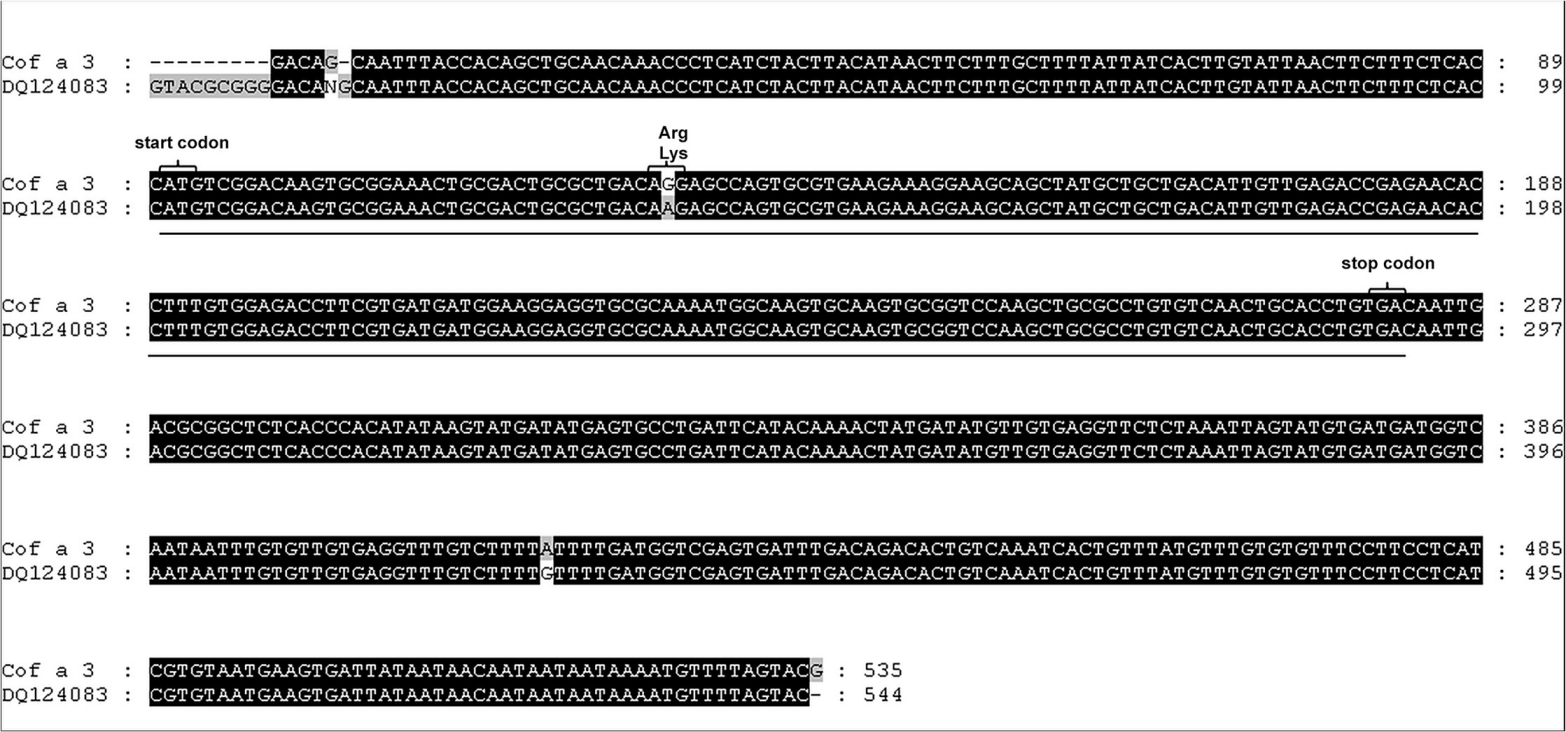
Sequence alignement of Cof a3 cDNA clone and its homologue NCBI DQ 124083. Homologous regions to the known coffee metallothionein described by Guzzo et al. ^14^ are highlighted in black. In position 138 and 426 of the protein forming open reading frame a polymorphism in the cDNA sequence is seen (underlined), in position 137–139 there is one single polymorphism in the amino acid sequence.

**Fig 3:**
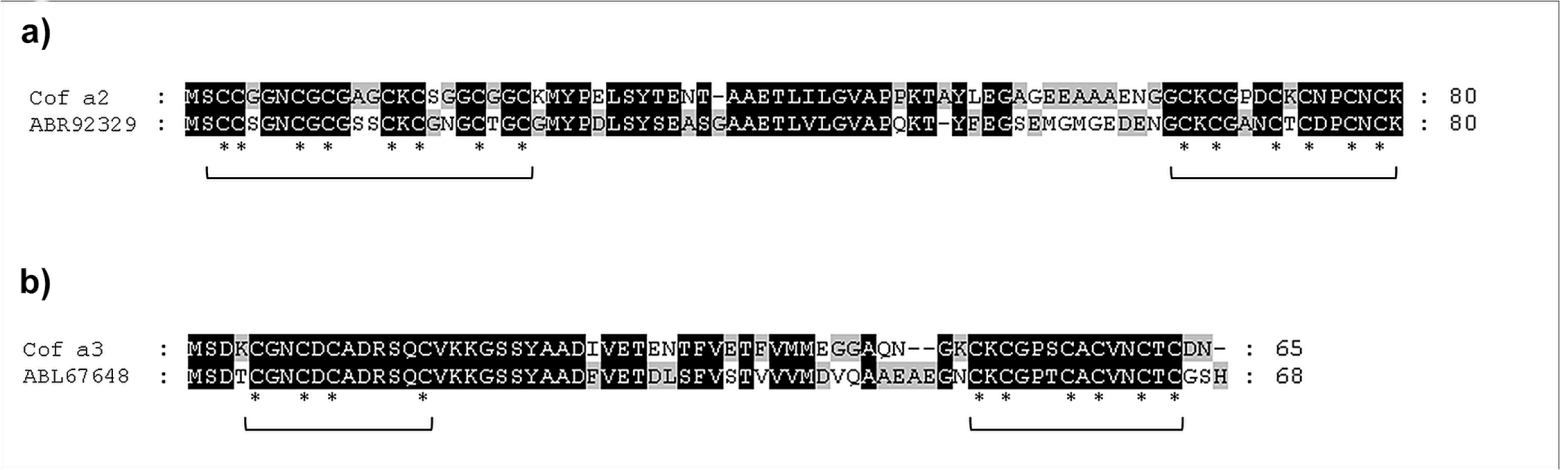
Alignement of the amino acid sequences. a: Cof a 2 and a *Salvia miltiorrhiza* metallothionein type II (ABR92329). b: Cof a 3 and a *Citrus hybrid* metallothionein type III (ABL67648) identified by BLAST search. Homologous regions with previously described metallothioneins are highlighted in black. The typical highly conserved domains of the cystein (*) arrangements of plant metallothioneins II and III are indicated by a square bracket.

Screening of the *Coffea arabica* phage display cDNA library retrieved a total of 62 IgE binding phages after three rounds of affinity selection. To identify the cDNAs encoding IgE-reactive epitopes, phagemids were extracted from affinity selected phages and subsequent insert cDNA sequencing was carried out. A BLAST search in the NCBI expressed sequence tags (EST) data base performed a 608 base pair (bp) cDNA insert, which we named Cof a 2, and a 563 bp cDNA insert, which we named Cof a 3. The first coffee metallothionein from immature *Coffea arabica* leaves was published by Moisyadi et al. in 1995 [13]. The Cof a 2 cDNA provided a nearly 95% sequence similarity over a region of 577 bp to a *Coffea arabica* cDNA clone (U11423) coding for a metallothionein (Fig 1). Likewise, Cof a 3 provided a nearly 99% similarity over a 535 bp region to a *Coffea arabica* cDNA clone (DQ124083) coding for a metallothionein type III (Fig 2). This EST from leaves was published by Guzzo et al. in 2009 [14]. Both cDNA clones include a complete ORF. The 243 bp long ORF of Cof a 2 is corresponding to a protein of 80 amino acids with a calculated molecular mass of 8,800 daltons. Comparison with entries in the NCBI protein data base revealed 69% amino acid identity with a *Salvia miltiorrhiza* metallothionein type II (ABR92329) (Fig 3A). Cof a 3 has a 198 long ORF, analogue to a 65 amino acids long and 7,150 daltons protein. A protein in the NCBI data base, corresponding for a *Citrus hybrid* metallothionein type III with 75% similarity (ABL67648) was also found (Fig 3B).

### Expression and purification of recombinant Cof a 2 and Cof a 3

**Fig 4:**
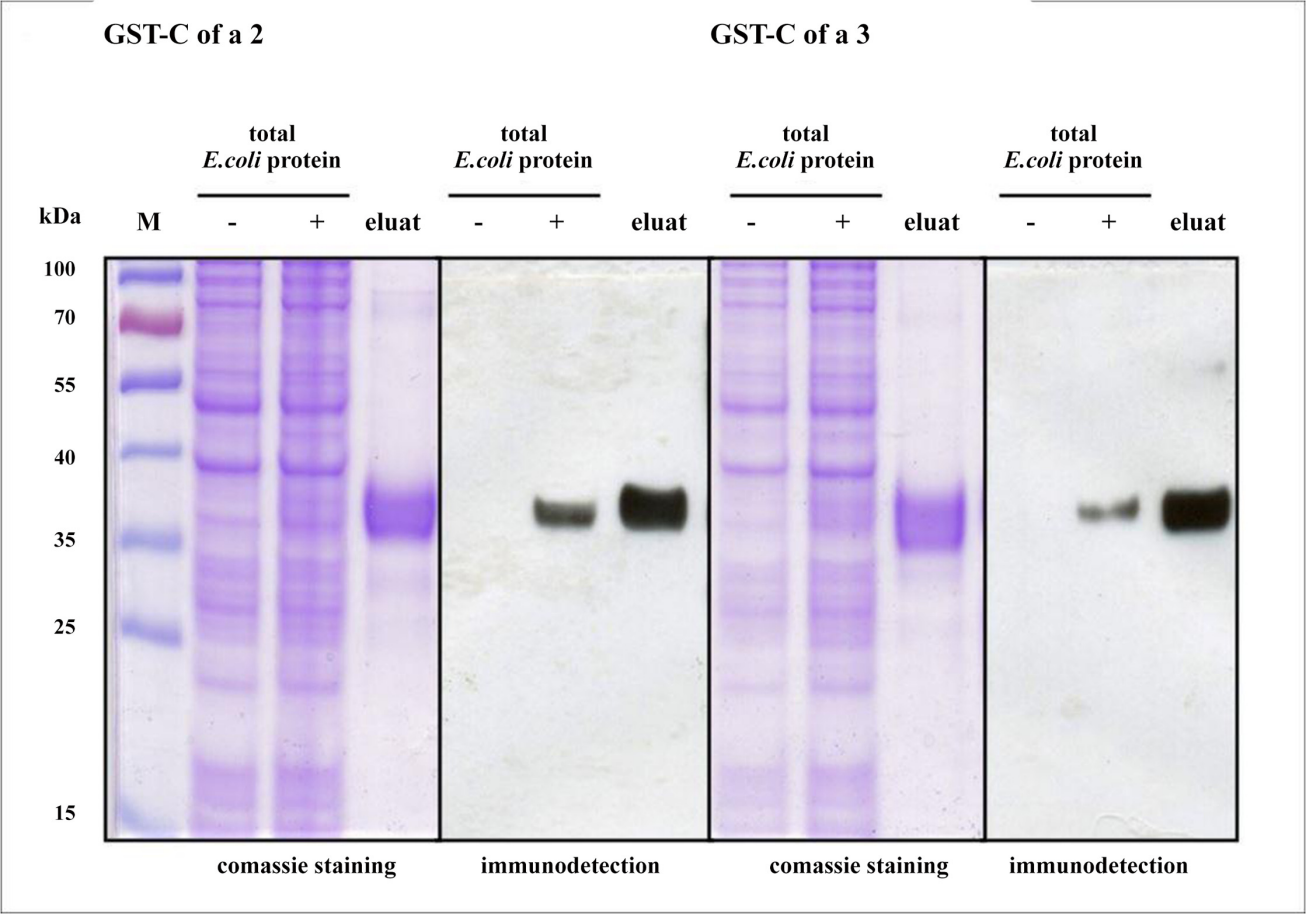
Purification and immunodetection of rCof a 2 and rCof a 3. Purification of *E*. *coli*-expressed rCof a 2 and rCof a 3 under native conditions. Proteins recovered by GST affinity cromatography were separated by SDS-PAGE (eluat). Total *E*. *coli* protein fractions before and after IPTG supplement are indicated by − and +; M: molecular wight marker. The first gel stained with coomassie brilliant blue, the second for immunodetection with anti His-Ab. Both gels ran together at same conditions.

The coding region of the cDNA insert was cloned into pET-41b(+) vector and expressed in *E*. *coli* strain BL 21 (DE3) as described in the methods section. A high amount of GST-Cof a 2-His and GST-Cof a 3-His fusion protein could be expressed in soluble form and purified using GST affinity chromatography. Examination of constructed proteins by SDS-PAGE and subsequent staining with Coomassie brillant blue revealed that GST-Cof a 2-His was strongly enriched as a band of the expected size of 35 kDa, and GST-Cof a 3-His protein by a size of 34 kDa. Purified proteins were verified by immunodetection using anti-His antibody (Fig 4 and S1, S2 and S3 Figs).

### Immunoreactivity of native coffee extract, rCof a 1, rCof a 2, and rCof a 3

Using commercial CAP analysis, sensitization to green coffee beans was found in 2/18 (11%) of workers with symptoms of allergic rhinitis and/or conjunctivitis under coffee dust exposure. ELISA screening showed specific IgE reactivity to rCof a 1, rCof a 2, and rCof a 3 in a total of 8 out of 18 (44%) sera of symptomatic coffee workers. One serum bound all recombinant allergens, the serum of another subject bound rCof a 1 and rCof a 2. Further six sera reacted to only one of the recombinant allergens. The two sera reacting to ImmunoCAP native green coffee (both CAP-class 3) did not react to any of the recombinant allergens (please note that these sera were not the ones used for the biopanning procedure). Total IgE levels were elevated (>100 kU/L) in 4 out of 18 (17%) sera, two of them reacted to native green coffee, the others did not react to any specific coffee allergen (see Table 1 and S1 Table).

To rule out that the GST tag (22 kDa), which was fused with the recombinant proteins, has IgE-binding capacity by itself, the sera were screened with purified recombinant GST. None of the sera bound GST.

**Table 1:** Clinical details of the study population and reactions to tested allergens.

	Subject no.	Age (yr.)	Sex	Work related complaints	Total IgE (kU/L)	rCof a 1	rCof a 2	rCof a 3	ImmunoCAP k70 (green coffee beans)
Coffee workers	1	52	m	c, r, ct	35.1	-	-	+	-
	2	41	m	c, r, ct	7.7	-	-	-	-
	3	49	m	c, r, ct	2.8	-	-	-	-
	4	53	m	r, ct	42.7	+	+	+	-
	5	46	m	c, u, ct	235	-	-	-	+
	6	47	m	c, u, ct	52.7	-	-	+	-
	7	42	m	c, r. ct	29.9	-	-	-	-
	8	54	m	c, u, ct	49.6	-	-	+	-
	9	41	m	c, r, ct	64.5	+	+	-	-
	10	54	m	c, r, ct	499	-	-	-	-
	11	47	m	r, ct	256	-	-	-	-
	12	33	m	r, ct	45.2	-	-	-	-
	13	54	m	c, r, ct	12.7	-	+	-	-
	14	34	m	c, r, ct	52	-	-	-	-
	15	45	m	c, r, u, ct	39.7	-	-	-	-
	16	29	m	c, r, u, ct	39.2	-	+	-	-
	17	38	m	c, r, ct	658	-	-	-	+
	18	48	m	c, r, u, ct	< 2.0	+	-	-	-
Control group	1	34	f	none	12.5	-	-	-	-
	2	50	f	none	72.8	-	-	-	-
	3	54	f	none	8.1	-	-	-	-
	4	25	f	none	40.9	-	-	-	-
	5	35	m	none	65.3	-	-	-	-
	6	38	m	none	17.1	-	-	-	-
	7	31	m	none	35.1	-	-	-	-
	8	29	m	none	48.3	-	-	-	-

m: male; f: female

c: conjunctivitis; r: rhinitis; u: urticaria; ct: chest tightness

-: no significant reaction; + positive reaction

All 8 control sera yielded negative results to purified rCof a 1, rCof a 2, rCof a 3 proteins, and to commercial ImmunoCAP with an extract of native green coffee beans.

After submitting the present data, the WHO/IUIS Allergen Nomenclature Sub-Committee has registered the novel coffee allergens as Cof a 2 and Cof a 3 [15–16].

## Discussion

Among workers exposed to green coffee dust in the coffee processing industry, a high prevalence of up to 50% of skin, eye, and/or airway symptoms has been described [2–4]. In view of a worldwide coffee processing of about 9 million tons per year [17], a relevant number of affected people has to be assumed. Beside irritative reactions to components of the coffee dust, allergic reactions, in particular work related type I-reactions to coffee dust, have to be considered to be the cause. Thus, there is a high need to understand the allergenic potential of green coffee for developing reliable diagnostic tools. Previously, we have identified the *Coffea arabica* class III chitinase Cof a 1 as an allergen in coffee workers exposed to the dust of green coffee beans [7], suggesting an antibody-mediated allergic genesis of the afflictions (type I hypersensitivity reaction).

Here we report two further coffee allergens. Since we were able to prove their relevant allergenicity, these allergens have been included in the IUIS Allergen Nomenclature as Cof a 2 and Cof a 3. They were identified as metallothioneins type II and III respectively. To date no single metallothionein has been shown to elicit type I hypersensitivity reactions yet. We were able to characterize the first two metallothioneins with potential to cause type I hypersensitivity reactions after inhalation. Metallothioneins are of stable structure due to their high amount of cysteine-rich regions. Cysteine is the only amino acid building disulfide bridges, and therefore cysteine rich regions are basically responsible for the stability of the three dimensional structure and thus could play a determinant role for allergenicity of proteins [18]. Low molecular weight of proteins has been associated with a stable configuration as well [18]. Due to the high amount of cysteine and the relatively small size of Cof a 1 with 32 kDa, Cof a 2 with of 9 kDa, and Cof a 3 with 7 kDa one would expect these proteins to have a stable configuration with high resistance to processing (such as roasting and cooking) and to digestion by digestive proteases. However, in the present study none of the sensitized coffee workers suffered from gastrointestinal symptoms after coffee intake. One reason for the discrepancy between the relatively high allergenicity by inhaling coffee allergens and the low allergen potential by oral intake might be specific allergen responses in different tissues [19]. On the other hand, these proteins might be not as stable and resistant as expected according to the structural characteristics mentioned above.

As we demonstrate, the only commercially available allergy tests based on native extracts of green coffee beans lacks sensitivity for diagnosing a considerable amount of affected coffee workers correctly. Our results indicate that the natural allergen extracts do not contain sufficient amounts of the chitinase III and metallothioneins II and III. The production of recombinant coffee allergens is relevant for the development of standardized, highly sensitive diagnostic tools for the determination of specific allergen patterns and for the development of allergen-specific immunotherapy [20]. For example, spiking the natural extract with recombinant coffee allergens could improve the diagnostics of coffee allergy. Despite the benefits of recombinant proteins in diagnostic procedures reduced IgE reactivity has to be considered always as a potential limitation when using them. Recombinant proteins may have been completely or partially unfolded (i.e. have an altered three dimensional structure) and might not be properly modified after translation. The lack of posttranslational glycosylations in *E*. *coli* can also have an impact on IgE-binding capacity [21]. This might be the reason why a proportion of exposed workers with allergic complaints to coffee dust did not react to any of the tested recombinant allergens. Besides this, it is also possible that the allergic complaints of these subjects are not elicited by allergens from coffee but from other components of the coffee dust, such as mould, mites, cockroaches etc.

Since we did not identify any major coffee allergens and it has to be assumed that there are numerous coffee proteins responsible for allergenicity, there is a need to expand the coffee allergen panel with allergens to be identified through further research.

All things considered, the allergens identified by us improve the diagnostic sensitivity, as we have shown here. Improved diagnostics of diseases related to coffee dust exposure are particularly relevant for the field of occupational health. The use of recombinant allergens can contribute to verify allergic diseases in coffee workers as occupational. Verifying the specific causal link between allergic disease and occupational coffee dust exposure is required in order to avoid adverse health and economic effects for affected employees.

In conclusion, we have identified two additional coffee allergens acting as possible inhalative allergens. We have shown that the previously described Cof a 1, as well as the here described Cof a 2 and Cof a 3 bind IgE from sera of symptomatic coffee workers and may be thus relevant for the specific diagnosis and/or therapy of inhalant coffee allergy.

## Supporting Information

S1 Fig Immunoplot rCof a 1, 2, 3. (TIF)

S2 Fig Immunoblot rCof a 1. (PPTX)

S3 Fig Immunoblot r Cof a 2, 3. (PPTX)

S1 Table ELISA rCof a 1, 2, 3. (XLSX)
