# Supplementary figures and images for: Identification of Two Metallothioneins as Novel Inhalative Coffee Allergens Cof a 2 and Cof a 3
Source: PLoS One. 2015 May 11;10(5):e0126455. doi: 10.1371/journal.pone.0126455 (PMC4427360; doi:10.1371/journal.pone.0126455)

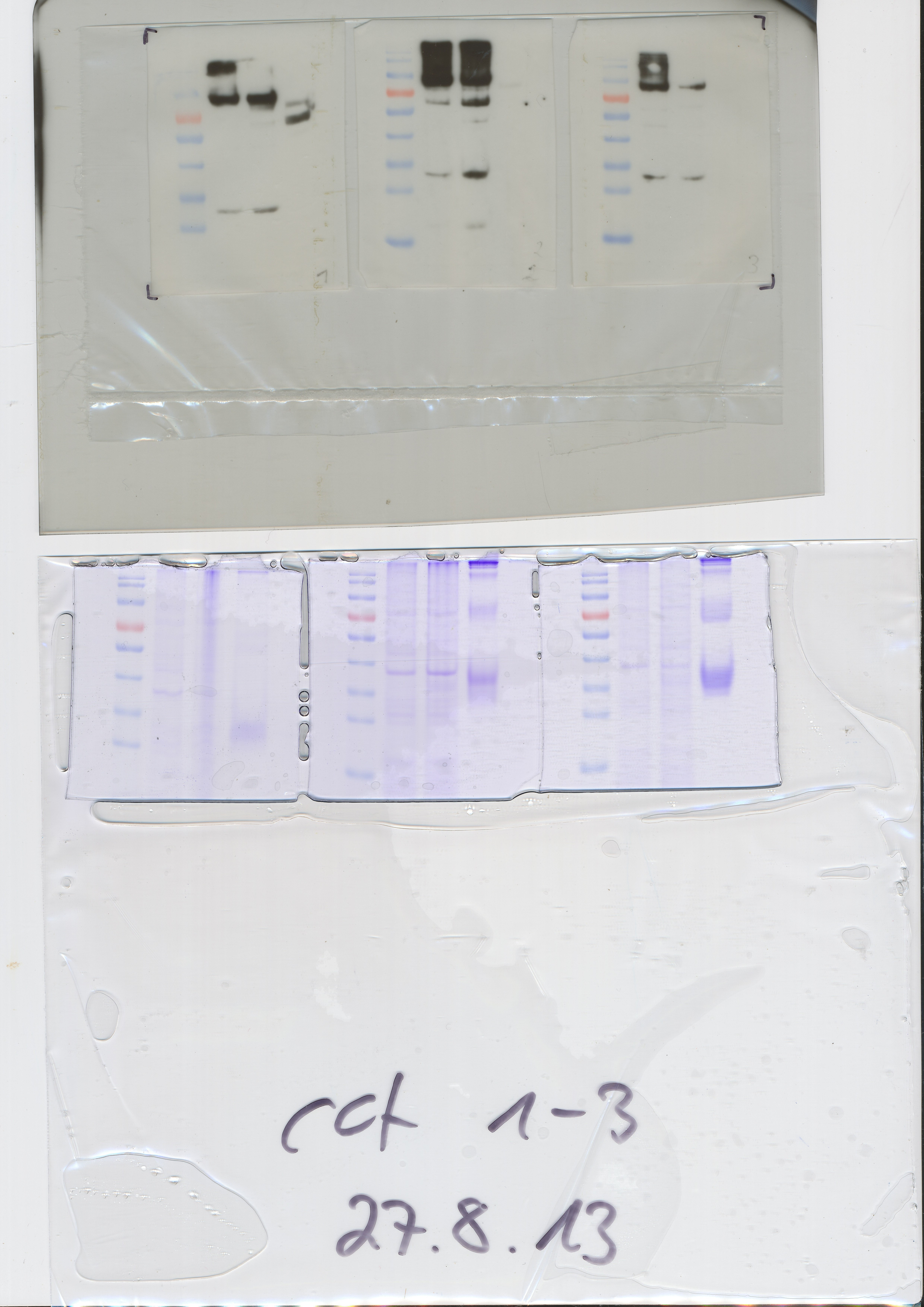

Supplement: S1 Fig — (TIF) [file pone.0126455.s001.tif]
