# Supplementary material for: Identification of Two Metallothioneins as Novel Inhalative Coffee Allergens Cof a 2 and Cof a 3
Source: PLoS One. 2015 May 11;10(5):e0126455. doi: 10.1371/journal.pone.0126455 (PMC4427360; doi:10.1371/journal.pone.0126455)
Supplement: S2 Fig — (PPTX) [file pone.0126455.s002.pptx]

## Slide 1
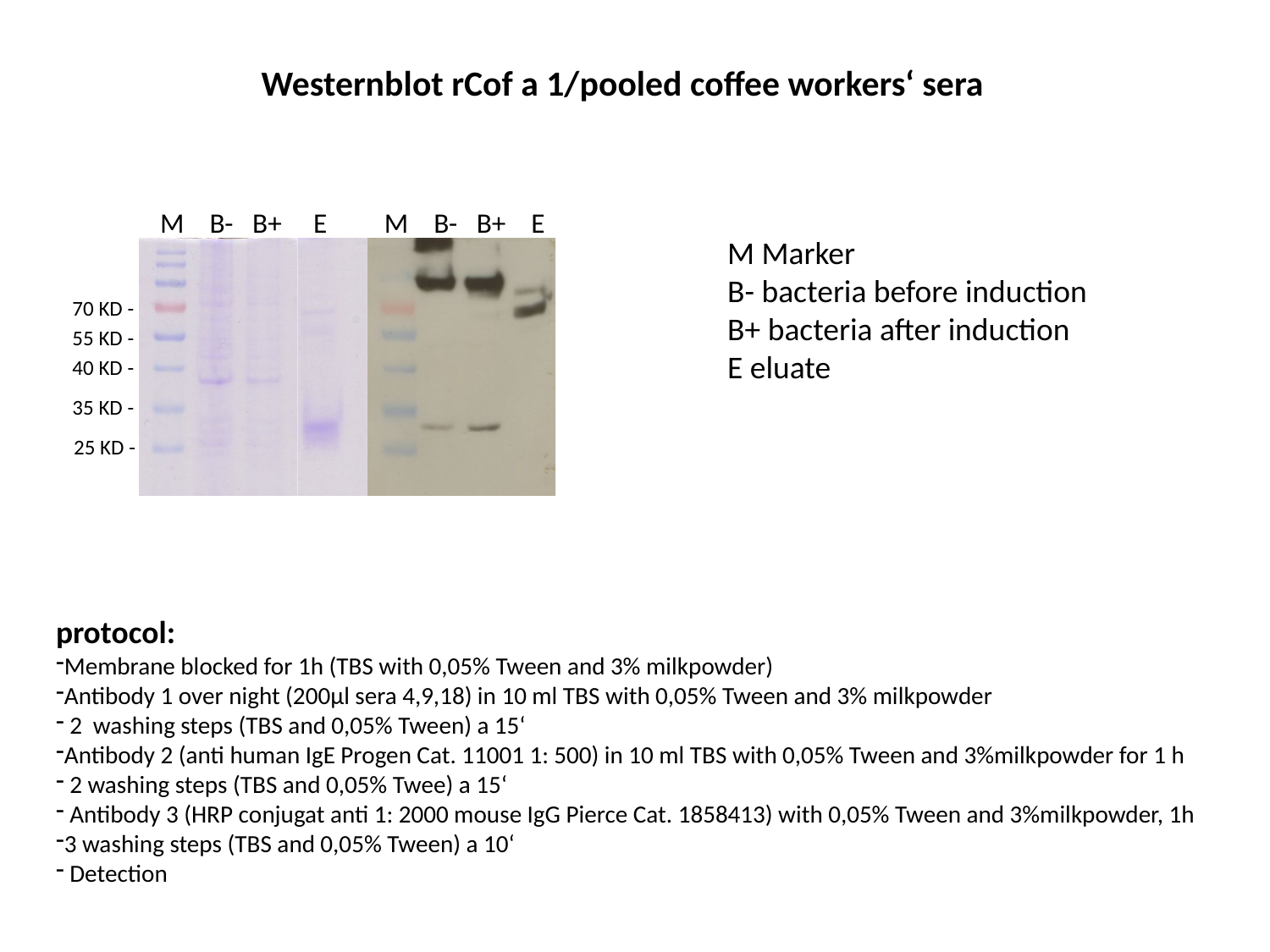

Westernblot rCof a 1/pooled coffee workers‘ sera
 M B- B+ E M B- B+ E
70 KD -
55 KD -
40 KD -
35 KD -
25 KD -
M Marker
B- bacteria before induction
B+ bacteria after induction
E eluate
protocol:
Membrane blocked for 1h (TBS with 0,05% Tween and 3% milkpowder)
Antibody 1 over night (200µl sera 4,9,18) in 10 ml TBS with 0,05% Tween and 3% milkpowder
 2 washing steps (TBS and 0,05% Tween) a 15‘
Antibody 2 (anti human IgE Progen Cat. 11001 1: 500) in 10 ml TBS with 0,05% Tween and 3%milkpowder for 1 h
 2 washing steps (TBS and 0,05% Twee) a 15‘
 Antibody 3 (HRP conjugat anti 1: 2000 mouse IgG Pierce Cat. 1858413) with 0,05% Tween and 3%milkpowder, 1h
3 washing steps (TBS and 0,05% Tween) a 10‘
 Detection
