# Supplementary material for: Identification of Two Metallothioneins as Novel Inhalative Coffee Allergens Cof a 2 and Cof a 3
Source: PLoS One. 2015 May 11;10(5):e0126455. doi: 10.1371/journal.pone.0126455 (PMC4427360; doi:10.1371/journal.pone.0126455)
Supplement: S3 Fig — (PPTX) [file pone.0126455.s003.pptx]

## Slide 1
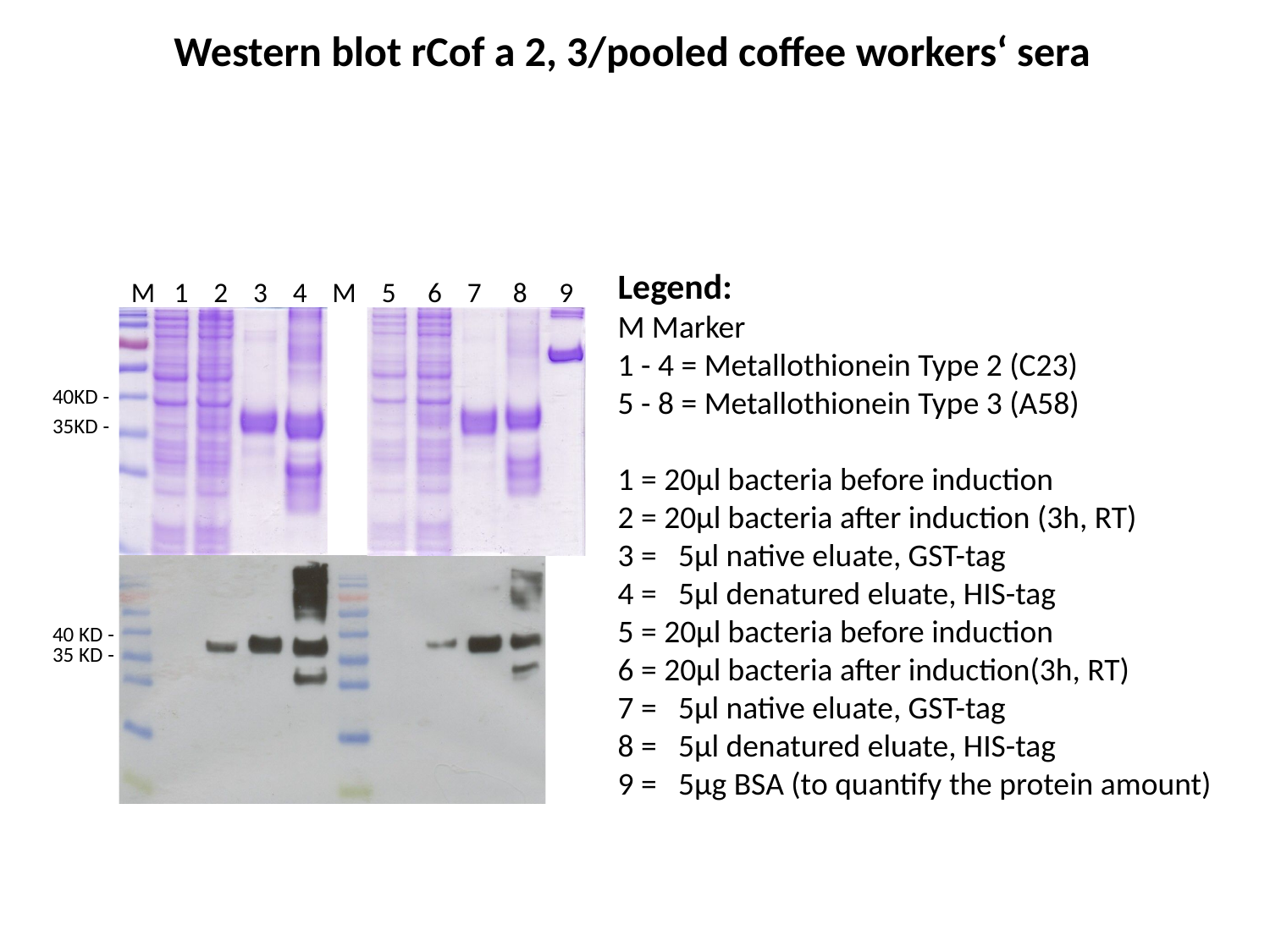

# Western blot rCof a 2, 3/pooled coffee workers‘ sera
Legend:
M Marker
1 - 4 = Metallothionein Type 2 (C23)
5 - 8 = Metallothionein Type 3 (A58)
1 = 20µl bacteria before induction
2 = 20µl bacteria after induction (3h, RT)
3 = 5µl native eluate, GST-tag
4 = 5µl denatured eluate, HIS-tag
5 = 20µl bacteria before induction
6 = 20µl bacteria after induction(3h, RT)
7 = 5µl native eluate, GST-tag
8 = 5µl denatured eluate, HIS-tag
9 = 5µg BSA (to quantify the protein amount)
 M 1 2 3 4 M 5 6 7 8 9
40KD -
35KD -
40 KD -
35 KD -
